# Medical Miscellanies

**Published:** 1817-08

**Authors:** 


### PART IV.

### MEDICAL MISCELLANIES.

### THE PORTE FEUILLE, No. VII.

OR,

DEPOT FOR DETACHED FACTS AND INTERESTING PHÆNOMENA OCCURRING IN THE PRACTICE OF THE FRIENDS OF THE MEDICO-CHIRURGICAL JOURNAL.

"Ore trahit quodcunque potest atque addit acervo."

#### *Aneurism of the Arteria Innominata.*

John Waller, ætat. 45, small stature, had worked for some years in the copper foundry in Portsmouth Dock Yard, where he was in the habit of using great exertion in pulling, dragging, &c. besides indulging in considerable quantities of malt liquors. About the beginning of 1816 he began to complain of various thoracic affections, of which he can give but a very confused account. Cough, pain under the sternum, head-ache, and pain in the right arm, were the most conspicuous symptoms. These were both preceded and accompanied by costiveness. He had applied to several medical men, and had been bled, with temporary advantage; but the complaint advanced, and early in 1817 *a diffused inflammatory swelling* appeared from the right side of the neck, down over the clavicle and along the right side of the sternum. He now applied to an able physician, who, by evacuations of all kinds, and cooling lotions, reduced the inflammation and symptomatic fever, and about the middle of March, the Reporter was requested by the physician to see the patient. The following was the state of the patient at this time: Cough; semipurulent expectoration; dysphagia and dyspnœa; inability to lie down; pulse pretty regular, and little accelerated; left carotid beats violently, and seems enlarged; right carotid can scarcely be felt. A small tumour is rising above the sternal portion of the right clavicle; the edge of which has pressed the trachea about an inch from the middle line, towards the opposite side. Upon accurate observation a pulsation could be ascertained, and it was pronounced aneurism. Bleeding; low diet; intestinal evacuations; cold applications continued. Complains of violent throbbing in his head, and pain and numbness in the right arm. The pulsation of the radial artery in this arm very indistinct. The heart pulsates regularly against the ribs, and does not seem affected, at least in any perceptible degree.

20th of April. The tumour has gradually but slowly pointed over the clavicle: its pulsations are now distinct; its parietes becoming attenuated. It has apparently ceased to encroach on the line of the trachea, which has become accommodated to the obliquity; and the difficulty of breathing and swallowing is not so much complained of as formerly; but the advance of the fatal catastrophe seems to bid defiance to the most rigid regimen.

12th. of May. For a week past the dysphagia had been so great that little or no food could be taken, and the dyspnœa and cough were very troublesome. He therefore was worn out for want of nutriment, and this day died.

*Sectio cadaveris.* The sternum was raised, the two clavicles sawn, and the whole bony front of the thorax removed without injuring the tumour, which was of a pyriform shape, rising by a peduncle from the arteria innominata. The upper part of the tumour in the neck was composed of twelve or fifteen layers of coagulum, apparently organized, yet separable. That part of the parietes of the sac, just above the clavicle, was as thin as peritonæum, and only covered by the integuments. The sac held about eight or ten ounces, and completely compressed the carotid of that side, and also the subclavian. The hole of communication with the arteria innominata admitted the fore finger. The heart was sound, the lungs adherent, but no actual disorganization.

The pathology of the above case is no wise singular; but it exemplified the difficulty of distinguishing internal aneurism at an early period. This man had been in a public hospital, and under several practitioners; it would be humiliating to the pride of medical science [perhaps salutarily so] to relate the *names* of the different diseases for which he was treated! Aneurism was not *suspected* till an external tumour appeared; and by some, was not admitted till after the day of the patient's death—nay, till two days after. This circumstance shews the value of *post mortem* examination.

MEDICO-CHIRURGUS.

---

### MISCELLANIES.

Quicquid agunt Homines.

### *Dr. Johnson's Explanation of a Passage relative to Dr. Balfour in the Observer No. I. Med. Chir. Journal, April* 1817.

Dr. Balfour in the Edinburgh Journal, *October* 1816, expressed himself thus:—"*It is the general opinion, and it has been sanc-*

tioned by the first medical authorities, that duration of inflammation in the extremities, for a considerable time, is equally conducive to the health of the body and to the vigour of the mind. This *conceit*, for it deserves no better name, tends more than all other considerations combined, to reconcile gouty patients to protracted sufferings." Dr. Johnson in the OBSERVER, No. 1 of this Journal, understood the word "*conceit*" as applied to the *profession*, and under *that* idea made the following comment. "When an individual, from such materials as the above two cases, draws a conclusion that *the general opinion of the profession*, sanctioned by the first medical authorities, is a mere *conceit*, it stamps that individual with the seal of presumption, so deep and so indelible, as to invalidate every antecedent and subsequent testimony from his pen." But it appears, (and indeed a more critical perusal of the passage will bear Dr. B. out) that the term *conceit* is only levelled at the *popular* opinion, sanctioned by "the first medical authorities." This, construction materially alters the position, and Dr. Johnsons censure no longer applies. Independently of this, however, Dr. Johnson never meant to question Dr. B's veracity by the foregoing comment, since, in the same paper, he expresses himself thus; "God forbid that I should entertain or express the slightest doubt of the fidelity or authenticity of Dr. Balfour's own cases." What he meant was this :—that when an individual contemns the general voice of the profession, and that on such slight grounds, his presumption warps his judgement, and "*invalidates*," that is, *weakens* the force of every thing he has or may write on *that particular topic*. Still Dr. Johnson, on reflection, thinks the language too severe, but declares upon his honour that he never meant, or harboured, the slightest *personal* allusion or hostility, in the said passage, towards Dr. Balfour. Dr. Johnson hopes that this declaration will clear any subsequent discussion of the subject from all appearance of rancour, and lead to a calm, dignified, and philosophic investigation on both sides.

---

### *To the Editors of the Medico-Chirurgical Journal & Review.*

GENTLEMEN,

HAD you refused to insert the Observer, No. 1, Article Gout, for the reasons you assign (in your *Notice to Correspondents*, No. 18,) for rejecting my Reply to that paper, you would have done yourselves honour; but having admitted the attack, you had no right to refuse me the hearing. It is better, however, that it has so happened, as the author of the Observer has since made himself known to me; and I trust to his pledged honour, that he will give such explanation of the obnoxious passages in his Paper, as will supersede the necessity of my noticing them. In this Paper, therefore, I shall strictly confine myself to the refutation of his objections to my practice in gout, to the elucidation of points in dispute, and to the corroboration of my former statements by additional facts.

In the first place then, the author of the Observer objects to my logic, when I say, " I see nothing in the nature of things why Compression and Percussion should not be as beneficial in Gout as in Rheumatism?" I say so still; for though the diseases are different, they yet are blended in a manner, with a frequency, and to an extent, which no other two diseases are ever observed to be. Such, indeed, is the similarity or sameness of their symptoms that, in many cases, it is difficult to draw the line of distinction, or to say, which of the diseases predominates in the same person, and at the same time. The diseases likewise affect the same parts, or parts of a similar structure ; and, their effects, if not in every instance the same, are in the great majority of cases extremely similar, whether we regard the appearance, or the impaired functions of the parts affected. Instances are not wanting, moreover, of rheumatic patients dying suddenly, as well as those of a gouty habit. Now, having been successful, beyond all precedent, in relieving and curing rheumatic affections combined with gout, by simply unloading and supporting the vessels of the parts affected, I submit it to the candid, if I am not justified in saying, " I see nothing in the nature of things why Compression and Percussion should not be as beneficial in Gout as in Rheumatism." If the professors of an experimental science were not to avail themselves of analogies, far less striking than that existing between rheumatism and gout, the advancement of the healing art would be slow indeed! A remedy that has been found eminently successful in cases of rheumatism and gout combined, must be applicable with the fairest prospect of success, in cases of simple gout; at least, I can see nothing in the nature of things why it should not. It is not on two cases, therefore, nor on ten cases, that I have " hastened to lay before the medical public," a new, simple, and expeditious mode of curing " gout." I have published two cases of gout separately, merely because they were well-marked instances of the acute form of the disease, and uncombined with any other complaint.

In the second place, my Reviewer asserts, " The first medical authorities do *not* attribute the salutary operation of a gouty paroxysm to the mere duration of inflammation in the extremities." *Not* indeed? I thought that, inflammation, and duration of inflammation for a considerable time in the extremities, constituted the very essence of a fit of regular gout. I imagined my Reviewer's only objection to my practice was, its interrupting and checking this process. Let us hear him for himself. " But the enlightened and observant pathologist sees, that a *constitutional* indisposition, a gouty diathesis, or whatever other term may be applied, is safely expended, as it were, at a distance from vital parts, during the confinement," &c. But how is this diathesis expended? by re-action, or the atony that succeeds? I presume my Reviewer will not say, by the latter; and if by the former, then the salutary operation of a gouty paroxysm is attributable to mere duration of inflammation. Thus, this "observant pathologist" denies a position in one breath, and affirms it in the next. He would have us believe there is a *real* difference where there is none, but in words; and, as I shall shew hereafter, that the difference between some things is trifling, where it is great and important. To Drs. Sydenham and Cullen I confidently appeal, as instances of first medical authorities sanctioning the opinion of the salutary effects of duration of inflammation in the extremities. "When the disease," says Dr. Cullen, "after having thus (509, 510) *remained for some time in a joint*, ceases entirely; it generally leaves the person in very perfect health, enjoying greater ease and alacrity in the functions of both body and mind, than he had for a long time experienced." *First Lines*, 511. But, why should I search for authorities? My Reviewer furnishes me with sufficient, in "the prophetic words of Cullen," *patience* and *flannel.*

In the third place, my Reviewer asks, if I "can so far blind myself by a favourite hypothesis, as to suppose that a trifling difference in the local application (cold or compression) can materially affect the principle of conversion?" I ask this gentleman, if he can so far blind himself to daily observation and experience, as to call the difference betwixt cold and compression *trifling?* Sudden vicissitudes of temperature are the sources of many of our most acute and dangerous diseases; compression and percussion, as I apply them, will obviate, but never can produce congestion internally. Cold applied to a part may paralyze it; compression restores and supports its tone. Cold is, in its very nature, repellent; compression and percussion conciliatory. Cold reduces the temperature of a part, by the chemical abstraction of heat; compression and percussion, by mechanically unloading the vessels and diminishing the resistance to the *vis a tergo* of the arteries that supply the part. It is impossible, indeed, that compression, regulated by the feelings of the patient and the circumstances of the case, can ever prove repellent; and I know not an agent more likely than percussion to excite the action of the extremities in cases of retrocedent gout.

On the principle of derivation and revulsion, I have, in innumerable instances, cured head-ache by percussion applied to the shoulders, to the whole of the spine, the true ribs, and superior extremities. In one case I removed, in the course of ten minutes, a dull obtuse pain in the right eye, accompanied with inflammation, by percussion applied to the shoulders. This patient's whole right side was affected with rheumatism, which was removed by one application of percussion, *a capite ad calcem.*

In fine, there is not "in the nature of things," the smallest reason to apprehend repulsion from the extremities to internal parts, from the practice I have recommended in gout. Could the contrary be shewn to me, either from reasoning, or matter of fact, I should be the first to warn the public against adopting it; well aware that a man degrades himself by persisting in an error, never by acknowledging it. My practice strikes at the root of no received doctrine, except that of "*patience and flannel;*" that is, *the doctrine of doing nothing*. My practice is not inconsistent with, but an improvement on, the most approved practice. By it the sufferings of patients are mitigated and shortened, the structure and functions are preserved entire and uninjured, which otherwise would be destroyed and lost; and all this, without increasing the risk of retrocession. Nothing can persuade me that perpetual lameness, that dislocated joints, secreting uric acid and carbonate of lime, the frequent consequences of violent, reiterated, uncontrouled inflammation in the extremities, can contribute to either the comfort, the safety, or longevity of any man. Nothing can persuade me that a gouty diathesis can be so effectually or so safely expended by confinement and inactivity, as by the due exercise of all the functions of the body.

In the fourth place, "Mark the end," says my Reviewer! "The publication of *cures*, leaving a veil over *consequences*, has been the cause of infinite evil in the medical world."—Leaving a veil over *consequences!* How could I conceal the "after-history" of my patients, were I ever so much inclined? How could I conceal it, had I ever so much reason? The *odium medicum* is not so inactive here, but that, had my practice been productive of evil, the monster that speaks with a hundred tongues would have proclaimed the matter. But I have no reason to conceal the "*after*-history" of my patients; and it is shortly this—Madame Rey is in France, where she never could have been but for the benefit she derived from compression and percussion. The other two are in perfect health of body and soundness of mind, and very indignant at my Reviewer's observations and suspicions.

In the fifth place, that in applying mechanical means to acute rheumatism and gout, I "tread on *tender ground*," I am well aware; and, therefore, in proportion as I find the ground tender, I tread lightly. I neither jolt, nor squeeze; nor, in any way whatever, torture my patients, as my Reviewer would insinuate. A remedy can never be objected to on the score of pain, which is regulated, in its application, entirely by the feelings of the patient. My success in the treatment of *acute*, as well as of *chronic* rheumatism, by mechanical, combined with other means, has already been such, as "to warrant the conclusion that friction, percussion, and bandages combined, are the most prompt, the surest, and the safest remedy for rheumatism, in all its stages, and in all the variety of forms in which it presents itself" *See my Treatise on Rheumatism*, page 243.—But should the truly liberal part of the profession, who rejoice in the multiplication of the means of combating disease, still remain sceptical on the subject, I would only say, *fiat experimentum*.

Having replied to the principal objections urged by my Reviewer, I proceed to relate concisely a few cases of simple gout, which I treated in the same way, and with the same success as those which have occasioned this controversy.

CASE 1. Mr. S. aged 47, of a plethoric habit, and accustomed to live fully, complained of pain at the flexure of the joint of one of his great toes; had from this cause been lame for eight years, and had swallowed an incredible quantity of *eau medicinale* and other specifics. I visited him only once, on the 2d of October last, and it was agreed he should wait upon me every day till better. I did not see him again, however; but, at the end of a week, he reported that the pain and lameness left him on that day I visited him, and "had not returned, no, not for a moment." At the end of two months, he sent me a verbal message that he remained quite well. At this moment (the distance of nine months) he walks as well as ever he did in his life.

CASE 2. A lady, 50 years of age, accustomed to live pretty fully, but a handsome, slender woman, was attacked, about the beginning of November last, with a pain at the flexure of the joint of the great toe, right foot. The complaint increased in severity for several days, till it reached a pitch altogether insufferable: she described it as more intolerable than the severest toothache; and it prevented walking, and rest in the night-time. I was sent for to try my plan of cure, by way of experiment, as she expressed it, rather than in the belief of being relieved by it. Two applications, however, of compression and percussion effected a complete cure, and the lady is now, and has been in perfect health ever since. This lady, who has read my Reviewer's observations, thinks she has a better chance of escaping internal disease, by her being enabled to take abundance of exercise in the open air, than if she had been confined for a long time to her chamber, and, for any thing she knows, rendered lame for life.

CASE 3. Mr. W. P. aged 30, applied to me, on the 6th of March last, with an affection similar to that just recited, but not so violent. It had existed a year, and affected his walking considerably. One operation, repeated at home by himself, removed the complaint, to the great joy of the patient, who was afraid of permanent lameness, and who piqued himself, and with reason, if a fine exterior is any reason, on his personal appearance.

CASE 4. Mrs. C. aged 55, has been long afflicted with gout in her feet and hands. Most of her fingers are swelled or distorted, or both. But what she most complains of is pain at the roots of the toes, shooting along the soles of her feet, which renders walking extremely painful and aukward, and seldom allows of sleep in the night-time for an hour together. On examination into this lady's condition, I could not promise to be of much advantage to her. There is a wide difference between recent cases, between cases of some months, or even a year or two's standing, and cases of ten, twelve, or twenty years' duration. In such cases as the latter, if the vessels of the joints affected are not in a great measure obliterated, their action, at least, is changed. In preventing such obliteration, or change of action, and in preserving the functions of parts entire, consists the great advantage of my mode of treating such complaints.— I was called to this lady on the 16th of May, and applied compression and percussion, in the usual way, for a short time. Next day, she informed me she slept the whole night without interruption, a comfort to which she had been long a stranger. She had a son-in-law, a physician, and a son, a surgeon, both resident in her house at the time; I therefore had no occasion to continue my visits. Her son informs me, that she continues to spend her nights agreeably; that when she gets damp feet, or walks too much, the pain returns in some degree, but that she controls it at pleasure by percussion, which produces a pleasant glowing sensation, attended with facility of motion and complete relief from pain.

Case 5. A gentleman, aged 47, of a fair complexion and very full habit of body; drinks wine excessively, and takes very little exercise. Was called to him on the 14th of April, when he informed me he was lame from a pain in one of his great toes; examined the part, and found the toe generally swelled and very painful at the flexure of the first joint. The patient now peremptorily demanded my opinion of the nature of the complaint, and I as decidedly told him it was gout; a piece of intelligence he was unwilling to hear. He submitted to an operation, and immediately walked much better. Incapable, however, though he moves in a very high circle, of comprehending how means so simple could have such beneficial effects, he did not require my longer attendance. In about a fortnight after I met him, when he informed me he continued well from the time I visited him, but that he had subsequently rubbed the parts with an unctuous substance, to which, he believed, he owed his cure! "Sir (said I), much good may your *grease* do you!"

WILLIAM BALFOUR.

*Edinburgh, 12th July,* 1817.

( To be continued. )

☞ *Dr.* JOHNSON, *the writer of* OBSERVER, No. I. *being scarcely convalescent from a severe illness, is unwilling to enter into controversy in the present state of his health and tenor of his mind. The Strictures of the* "OBSERVER," *and the Reply of Dr.* BALFOUR, *are now fairly before the Public, and* TIME, *which is seldom wrong in its judgments, will determine between them. Dr. J. has only to reiterate his disavowal of all* personal *opposition to the peculiar opinions and practices of Dr.* B. *but he still claims the privilege of adhering to his former sentiments.*

---

*Alibert's Anatomical and Chemical Examinations of Dermoid Diseases.*—There is in general so little danger in the progress and results of cutaneous diseases, that any opportunity for anatomical investigation after death, seldom occurs. Such opportunities could only be found among the very great number of patients with these diseases, which are received into the extensive hospital of St. Louis at Paris; of these, "Alibert," the chief medical officer of that establishment, has availed himself; and in the article "Dartre," in the Dictionnaire des Sciences Medicales, has given the three following cases.

First. A soldier, aged 35 years, while serving in the cavalry, had a humid squamous eruption (*herpes squammosus madidans*) on the left buttock, which was greatly aggravated by the duties of a campaign. It extended so considerably, that the paetient when he reached Paris, was in a deplorable state; his legs swoln, general emaciation, and constant fever. In this condition he was received into the hospital of St. Louis. Although many remedial means were employed, no favourable change was observed in the symptoms. A month was passed in distress and languor: the patient became frightfully wasted. One day, in the act of respiring, he felt a most acute pain, and died almost immediately. On examination, that part of the teguments occupied by the eruption, was found thickened and gangrenous in all points; the cellular membrane was laden with yellow fat. On opening the thorax, the right lung was found inflamed, and adherent to the ribs. The alterations observed in the abdominal cavity were more strongly marked; the liver was uncommonly large and converted into fat; the intestines presented evident traces of chronic inflammation.

Second. A female, aged 20 years, who obtained her living by embroidering, had been affected from her infancy with a circular furfuraceous eruption (*herpes furfuraceus circinatus*) which shewed itself in patches on the face, the neck, about the ears, on the breast, the exterior of the fore-arm, and about the articulations of the elbows. The abdomen, the thighs, the knees, and the legs, were also similarly affected. The patient's aspect was hideous. From the employment of some repelling remedies, which she procured from a quack, the eruptions quickly disappeared; but soon after there was a suppression of the menses, with difficult respiration, extreme anxiety, and almost imperceptible pulse. In this state she continued forty days, when the inferior extremities became anasarcous, the face puffy, &c. She died from suffocation.

On opening the body, the following alterations were noticed: The pleura of a livid red colour, and much thickened; its inner surface covered with an albuminous deposition, easily removable with the handle of a scalpel; hydrothorax. On the right side, the fluid was sero-purulent, the colour apple green; on the left side, the fluid was serous and yellow; the lungs diminished in size, and pushed towards the anterior part of the chest—both adhering to the pleura; dropsy of the pericardium; the heart preternaturally large; in both ventricles several clots of black blood; the right more dilated than the left; no lesion was discernable in the abdominal viscera, but they were floating in a vast collection of serous fluid. The brain was soft, and its vessels gorged with black blood.

Third. Josephine Brugnon, 18 years of age, died, in a deplorable reduced state, from an eruption, which for a time presented no particular character beyond that of a yellow incrustation *(herpes crustaceus flavescens)*, but subsequently spread and assumed the phagedenic character: this fatal change was probably attributable to various distresses which she had experienced, and to the immoderate use of spirituous potations. On the first appearance of the eruption, the parts it occupied were examined: they were of a purple red colour, covered with small pustules containing a turbid thick fluid, the concretion of which gave rise to the formation of the crusts. The circumference of the mouth was wholly covered with similar incrustations; but their colour was a blackish grey, approximating them in appearance to those produced by the phagedenic "dartre." Thus, it was on this part that the conversion of the disease took place. The eruption had so extended in eighteen months, that all the upper lip, the cartilages and proper bones of the nose, were successively destroyed. The poor sufferer lingered for some time, and daily wasted; her skin acquired an extreme dryness, and resolved itself into a farinaceous matter. The gums and mucous membrane of the mouth, assumed a white appearance: at length she died, and the examination of the body was proceeded upon with the minutest attention. The following appearances were remarked:

*External phœnomena.* The teguments, as has been already noticed, were dry, wrinkled, and of a dirty grey cadaverous hue; the muscles appeared emaciated through their whole extent; the alæ of the nose had disappeared, as had also the septum and the bones which formed the nasal fossæ.

*Internal phænomena.* The abdomen was in a natural state, but entirely divested of fat; the peritoneum was thickened and spongy at the umbilical region, and through its whole extent it was studded with numerous hard yellow granulations of an irregular form. There was a considerable effusion of serous fluid; and the mucous membrane of the digestive canal was pale, and appeared as if it had been macerated: the liver was larger, and more compact than ordinary, and of a dingy yellow colour; in the gall bladder was observed some blackish, viscid, stringy bile; the pancreas was more developed than in a natural state; the spleen possessed more firmness than is usually found, but neither the bladder nor their kidnies had undergone any alteration; there was no observable lesion of the womb; the Fallopian tubes were ulcerated at their extremity, and the tissue of the ovaria was slightly deteriorated. On opening the thorax, the pleura and lungs were found in a healthy state, but there was a deposition of a white albuminous matter on their surface; there was no effusion of any kind; the heart was shrunken, and void of blood. The mucous membrane of the larynx, pharynx, and œsophagus, had a macerated appearance,

In giving the results of these examinations, says Alibert, I have no hope that they will throw any great light on the nature, the diagnosis, the seat, the causes, or treatment of these complaints; but I would at least wish to shew, that similar investigations ought not to be neglected, as they may lead to the discovery of more instructive and interesting facts than we at present possess.

*Chemical examination.* Chemistry, observes the author, is a species of material dissection, by which important phænomena may be developed. An exact and comparative analysis of every morbific virus to which the dermoid system is a prey, might perhaps afford considerable advantages to the progress of pathology. Some years since, continues our author, I sent a large quantity of *dartrous* scales and incrustations to M. Vauquelin's laboratory. These are the results which were obtained from them:

*The scales.* 1st, albumen; 2dly, animal mucilage; 3dly, muriate of soda; 4thly, sulphate of soda; 5thly, free phosphoric acid; 6thly, phosphate of lime.

*The incrustations.* 1st, albumen; 2dly, animal mucilage; 3dly, muriate of soda; 4thly, sulphate of soda; 5thly, sulphate of lime; 6thly, carbonate of lime.

Thus, the only difference found between these morbific substances was, that the scales contained free phosphoric acid, and no carbonate of lime; while the incrustations afforded no acid, but contained the carbonate of lime. L.

---

At a Quarterly Court of Assistants of the Royal College of Surgeons, held on the 15th of July, Sir James Earle was elected Master, and George Chandler and Thomas Keate, Esquires, were elected Governors of the College for the year ensuing.

---

NEW PUBLICATIONS.

Professor Orfila, author of the celebrated Treatise on Animal, Mineral, and Vegetable Poisons, has in the Press, an elementary work on Chemistry: it is expected that an English Translation will appear soon after the publication of the Original. From the situation of Dr. Orfila, as Teacher of the Science of Chemistry in Paris, together with his correspondence with Professors in this and other countries, we may suppose that this chemical work will contain all the modern discoveries which have been made in the science; and, as such, be rendered an useful book for Students.

Dr. Bancroft has in the Press, and nearly ready for publication, A Sequel to his Essay on Yellow Fever.

In the Press, and speedily will be published, The History of Vaccination. By James Moore, Surgeon.

In the Press, and nearly ready for publication, the Second Edition, corrected and enlarged, of Practical Observations on the Cure of the Gonorrhœa Virulenta in Men. By Thomas Whately, Surgeon.

FOREIGN BOOKS RECENTLY PUBLISHED.

Le Regne Animal distribué d'apres son Organization; par G. Cuvier, &c. avec figures. Tom. iv. octavo.

Nosologie Naturelle, ou les Maladies du corps Humain, distribuées par familles. Par Alibert. Tom. ii. quarto.

---

DEATHS.—Lately, in the meridian of life, after a long and severe illness, John Pedley, Esq. Surgeon, of Wednesbury, Staffordshire.—At an advanced age, Thomas Salt, Esq. formerly Surgeon of Litchfield, and father of the celebrated Abyssinian Traveller. He was a man of sound intellect, and deep and accurate observation; and had practised for many years with great success in Lichfield. Dr. Palmer pays this last tribute of respect to the memory of his early preceptor.

---

